# Molecular Motions as a Drug Target: Mechanistic Simulations of Anthrax Toxin Edema Factor Function Led to the Discovery of Novel Allosteric Inhibitors

**DOI:** 10.3390/toxins4080580

**Published:** 2012-07-31

**Authors:** Élodie Laine, Leandro Martínez, Daniel Ladant, Thérèse Malliavin, Arnaud Blondel

**Affiliations:** 1 Laboratoire de Biologie et de Pharmacologie Appliquée, Ecole Normale Supérieure de Cachan, 61, avenue du Président Wilson, 94235 Cachan cedex, France; Email: elodie.laine@lbpa.ens-cachan.fr; 2 The Molecular Biotechnology Group, Institute of Physics of São Carlos, University of São Paulo, Av. Trabalhador Sãocarlense, 400, 13566-590 São Carlos, SP, Brazil; Email: leandro@ifsc.usp.br; 3 Unité de Biochimie des Interactions Macromoléculaires and CNRS UMR 3528, Département de Biologie Structurale et Chimie, Institut Pasteur, 28, rue du Dr. Roux, 75724 Paris Cedex 15, France; Email: ladant@pasteur.fr; 4 Unité de Bioinformatique Structurale and CNRS UMR 3528, Département de Biologie Structurale et Chimie, Institut Pasteur, 25, rue du Dr. Roux, 75724 Paris Cedex 15, France; Email: terez@pasteur.fr

**Keywords:** anthrax, Edema factor, molecular modeling, virtual screening, allostery, transition path

## Abstract

Edema Factor (EF) is a component of *Bacillus anthracis* toxin essential for virulence. Its adenylyl cyclase activity is induced by complexation with the ubiquitous eukaryotic cellular protein, calmodulin (CaM). EF and its complexes with CaM, nucleotides and/or ions, have been extensively characterized by X-ray crystallography. Those structural data allowed molecular simulations analysis of various aspects of EF action mechanism, including the delineation of EF and CaM domains through their association energetics, the impact of calcium binding on CaM, and the role of catalytic site ions. Furthermore, a transition path connecting the free inactive form to the CaM-complexed active form of EF was built to model the activation mechanism in an attempt to define an inhibition strategy. The cavities at the surface of EF were determined for each path intermediate to identify potential sites where the binding of a ligand could block activation. A non-catalytic cavity (allosteric) was found to shrink rapidly at early stages of the path and was chosen to perform virtual screening. Amongst 18 compounds selected *in silico* and tested in an enzymatic assay, 6 thiophen ureidoacid derivatives formed a new family of EF allosteric inhibitors with IC50 as low as 2 micromolars.

## 1. Introduction: Edema Factor, a Target of Choice to Fight Anthrax?

### 1.1. Virulence Factors

The virulence of *Bacillus anthracis* is mediated by the cooperative and synergistic action of three main proteins: a cell-binding protein, the protective antigen (PA), and two enzyme components, the lethal factor (LF) and the edema factor (EF). PA promotes EF and LF translocation in the cytosol of infected cells, particularly macrophages, where the two enzymes perform their damage-inducing processes, allowing bacteria to evade the immune system [1]. LF is a zinc-mediated metalloprotease, which cleaves MAP kinases and possibly other substrates. This impairs cell signaling, and results in the induction of apoptosis.

EF, the main focus of the present study, is an adenylyl cyclase. It is activated within the eukaryotic target cells upon association with the host ubiquitous calcium sensing protein, calmodulin (CaM). The formation of the complex induces a large conformational change for EF. The activated toxin greatly increases the level of cAMP in the cells, and thus disturbs major intracellular signaling pathways ultimately leading to severe cellular dysfunction. More recently, EF has been shown to display other nucleotidyl activities, which could also contribute to toxic effects [2]. EF has been described to play central roles in the immune response impairment [3], during the infection [4] or the associated septic choc [5,6].

### 1.2. Bioterrorism Threat

The ability of the bacterium to form very resistant endospores that can survive for decades in the soil and spread easily though water and air makes it a potential biological weapon [7,8]. In addition, the spores are relatively easy to produce, so that anthrax constitutes a microorganism of choice for bioterrorism. Consequently, it appears important to investigate ways to rapidly block anthrax infection and toxic detrimental effects, while at the same time ensuring and improving administration convenience and patient compliance [9,10].

### 1.3. Prevention and Treatments

Several classical therapeutic approaches can be used to fight anthrax disease [11]. Since the original vaccine trials by Louis Pasteur in 1881, an attenuated Stern strain is still successfully used as a live avirulent vaccine in livestock [12]. Numerous human vaccines based on PA have been developed in the 1960s. However, due to intensive dosing regiment and high reactogenicity, these vaccines are reserved for high-risk population treatment only [13]. Recently, anthrax capsules or whole inactivated spores have been incorporated in newly developed vaccines [14,15]. Efforts have also been made to combine smallpox and anthrax vaccines [16].

Antibiotics (penicillin, doxycycline and ciprofloxacine) [17] lead to rapid recovery if administered very early in the disease development. Although the number of naturally antibiotic-resistant strains is low [18], the emergence of resistance due to treatment exposure or *in vitro* engineering is a clear concern [19,20,21]. The latter observation and the existence of the non-natural bioterrorism threat call for more efficient and rapid ways to block anthrax infection and toxicity.

### 1.4. Targets

In that context, the anthrax toxins, which bear key virulence activities, can be regarded as privileged drug targets to fight the disease by means of selective inhibitors. Most of the drug design efforts have been focused on PA and LF so far. However, recent experimental evidences showing the crucial roles of EF in the repression of the immune response [3], in the infection [4], and in the septic shock [5], suggest that EF should be a valuable target for the design of small molecule inhibitors.

### 1.5. Structural Data

**Figure 1:**
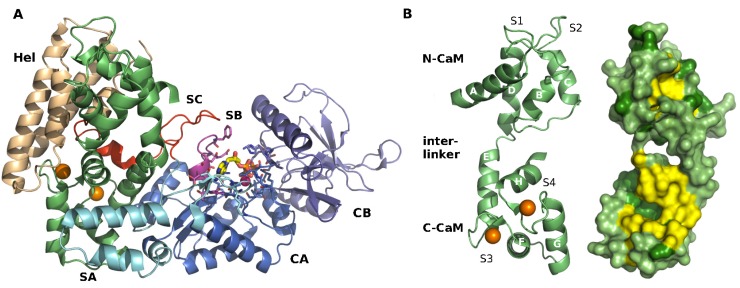
X-ray crystallographic structure of the EF-CaM complex [23]. (**A**) EF and CaM are displayed in cartoon representations. Calmodulin (CaM) is in green lime and loaded with two calcium ions (*spheres in orange*). The helical domain Hel of EF is in wheat, the CA and CB domains that form the catalytic core of EF are in marine and purple respectively. The three switches SA, SB and SC are colored in cyan, magenta and red. The catalytic residues and the ligand 3'-deoxy ATP are drawn in sticks and the Yb^3+^ ion is drawn as a magenta sphere; (**B**) Cartoon and surface representations of CaM structure are displayed from different views. On the left, the eight helices of CaM are labeled: A, B, and C in N-CaM; D and E in the interlinker; and F, G, and H in C-CaM. Calcium-binding loops S1 and S2 in N-CaM, S3 and S4 in C-CaM are indicated. Two Ca^2+^ ions are bound to S3 and S4 (*spheres in orange*). On the right, CaM hydrophobic patches as defined by Yang *et al*. [30] are colored in yellow and the other hydrophobic residues are colored in forest green.

During the last ten years, to support fight against anthrax, a large effort in structural biology has been undertaken to better understand the biophysics of anthrax toxins translocation across cell membranes, activation in the cytoplasm and enzymatic activity. In particular, Edema Factor (EF) has been the subject of intensive investigation using X-ray crystallography (Table 1) in the laboratory of Wei-Jen Tang (Ben May Department for Cancer Research, University of Chicago). Once the appropriate crystallographic conditions had been determined [22], the first structures of EF isolated (1K8T) or in complex with CaM (1K90, 1K93) were solved at 2.90–2.95 Å resolution and published [23] in 2002. These structures pictured an unexpected mode of action for calmodulin (CaM). CaM and EF form a tight complex in which CaM adopts an extended form, while EF conformation strongly differs from its CaM-free form (about 9.5 Å RMS difference, Figure 1). NMR measurements [24] clarified the influence of calcium on the formation and stability of the complex. In 2005, Shen *et al.* published additional EF-CaM complex structures at 3.0 Å resolution. It included the *N*-terminal PA binding domain of EF as well as 2, 3, or 4 bound calcium ions (1XFX, 1XFY, or 1XFV respectively). However, these structures collected in different conditions did not reveal any major variation, and thus, crystallographic studies could not explain the influence of calcium on the complex stability.

**Table 1:** List of PDB structures containing the Edema Factor.

PDB id	Reference	Description
1K8T	[23]	EF in free inactive form
1K90	[23]	EF in complex with CaM and 3'-deoxy-ATP
1K93	[23]	EF in complex with CaM
1LVC	[25]	EF in complex with CaM and 2'deoxy 3' anthraniloyl ATP
1PK0	[26]	EF in complex with CaM and PMEApp *^a^*
1S26	[27]	EF in complex with CaM and 5' met-ATP
1SK6	[28]	EF in complex with CaM and cAMP, PPi *^b^*
1XFU	[29]	EFΔ64 *^c^* in complex with CaM
1XFV	[29]	EF in complex with CaM and 3'-deoxy-ATP
1XFW	[29]	EF in complex with CaM and cAMP
1XFX	[29]	EF in complex with CaM, 10 mM calcium
1XFY	[29]	EF in complex with CaM
1Y0V	[29]	EF in complex with CaM and PPi

*^a^* Adefovir diphosphate; *^b^* pyrophosphate; *^c^* an Edema Factor (EF) truncation mutant where the sequence 33–63 was removed.

### 1.6. EF Inhibitors

In the past decade, intensive efforts have been devoted to the design of small molecules able to inhibit EF and that could be used as tools to study anthrax toxin effects on cells and could also be potential leads toward anthrax therapeutics. Recent works have highlighted the importance of EF in host-pathogen interactions and have further validated the idea that EF may effectively be a valuable therapeutic target to fight against the development of anthrax [4].

**Figure 2:**
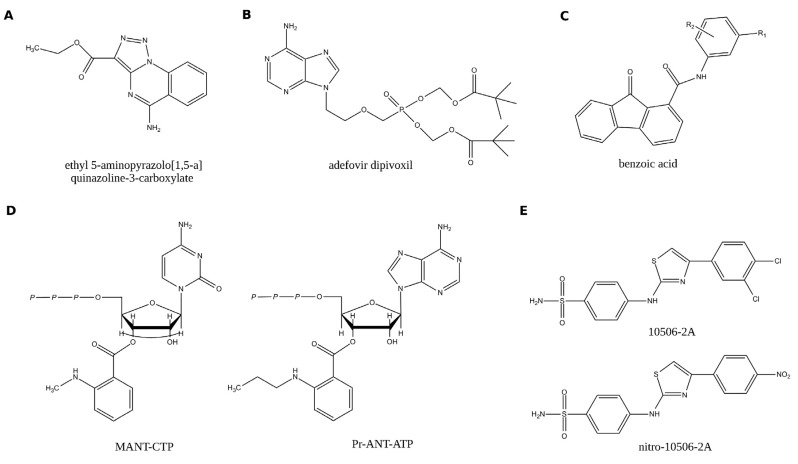
Examples of potent inhibitors of EF toxin component. Inhibitors in panel A to D were identified by targeting EF active site. (**A**) ethyl 5-aminopyrazolo[1,5-a]quinazoline-3-carboxylate, [32]; (**B**) (9-[2-[[bis[(pivaloyloxy)methoxy]phosphinyl]methoxy]ethyl]adenine; or bis-POM-PMEA, [26]; (**C**) 3-[(9-oxo-9H-fluorene-1-carbonyl)-amino]-benzoic acid, [33,34]; (**D**) MANT-CTP and propyl-ANT-ATP, [35]; (**E**) 4-[4-(4-dichloro-phenyl)-thiazolylamino]-benzenesulfonamide (10506-2A) was first identified in a cell based assays and then selected for its ability to block EF–CaM binding. 4-[[4-(4-nitrophenyl)-2-thiazolyl] amino]-benzenesulfonamide (nitro10506-2A) was identify as a non-toxic derivative of the former [36]. They appeared to bind the helical domain of EF, thus being allosteric as the compounds of the TUA family, which will be described below (Figure 5, and ref. [37]).

In principle, three major modes of action can be conceived for small molecules acting as inhibitors of EF, each targeting a distinct step along the EF intoxication process, namely (i) EF entry into target cells; (ii) EF activation by CaM and finally; (iii) EF enzymatic activity. We will not further describe here the first mode, as the corresponding molecules are mainly directed toward the PA component which is responsible for the internalization of EF (and LF) into eukaryotic cells (see [31]). Most of the known EF inhibitors target the active site (Figure 2A–D) and only two inhibit the EF/CaM interaction (Figure 2E), as summarized below.

#### 1.6.1. Inhibitors Targeting EF Active Site

Following the description of EF/CaM structures, the first discovery of EF inhibitors using structure-based approach was reported in 2003 by the team of W.-J. Tang [32]. They were directed towards the catalytic site of EF and the identification of ATP competitors. About 200,000 molecules (from the Available Chemical Directory) were docked in multiple orientations and conformations into the ATP binding site (Protein Data Bank code 1K90). About twenty molecules with high scores were selected for further experimental characterization. Among them, few were shown to specifically inhibit EF in vitro and to block the cytotoxicity of EF (+PA) toward eukaryotic cells. These authors thus identified a family of quinazoline compounds which selectively inhibited EF, with *K_i_* values of 20–50 µM for the best one (ethyl 5-aminopyrazolo[1,5-a]quinazoline-3-carboxylate, Figure 2A), without inhibiting mammalian adenylyl cyclases (MAC(s)). Interestingly, these molecules also inhibited the related CaM-dependent adenylate cyclase toxin, CyaA, produced by *Bordetella pertussis* the causative agent of whooping cough.

The same team later examined a series of nucleotide analogues that had been previously clinically approved for a variety of viral infections (and would thus exhibit favorable pharmacological properties). They found that adefovir dipivoxil (9-[2-[[bis[(pivaloyloxy)methoxy]phosphinyl]methoxy]ethyl]adenine; or bis-POM-PMEA, Figure 2B), a drug clinically approved to treat chronic infection of hepatitis B virus, can efficiently block the EF induced cAMP accumulation in mammalian cells and the resulting pathological effects such as alteration of cytokine production in macrophages [26]. This drug is converted inside the cells by cellular kinases into a diphosphate derivative, PMEApp, a non-cyclizable ATP analogue that binds with high affinity of the catalytic site of EF as revealed by X-ray crystallography. Kinetic analysis revealed that PMEApp is a highly potent competitive inhibitor (*K_i_* = 27 nM) of EF. Since this original report, adefovir dipivoxil has been used to explore the role of EF in anthrax pathogenesis [3,4,38].

To identify non-nucleotide inhibitors of EF, Chen *et al*. [33] used a structure-based method in which a 3D-pharmacophore, fitting the EF active site, was constructed from fragments. A small set of compounds exhibiting the best docking scores in virtual screening were tested in a cell-based assay for their ability to reduce cAMP release by cells treated with EF/PA. Four compounds having different molecular structures exhibited IC50 values in the low micromolar range in these assays [33]. More recently, the same team used molecular docking to predict improvements in potency and solubility of one of these molecules (3-[(9-oxo-9*H*-fluorene-1-carbonyl)-amino]-benzoic acid, Figure 2C). A structure-activity relationship (SAR) analysis of a number of variants yielded few derivatives with superior pharmacological properties as compared to the initial lead compound [34].

The group of R. Seifert also developed potent and selective inhibitors of EF (as well as of *Bordetella pertussis* CyaA) based on various purine and pyrimidine nucleotides modified with *N*-methylanthraniloyl (MANT)- or anthraniloyl (ANT) groups at the 2'(3')-*O*-ribosyl position. MANT-CTP and propyl-ANT-ATP were the most potent EF derivatives characterized (Figure 2D, [35]). In *in vitro* assays, these compounds inhibited EF competitively with *K_i_* of 100 nM and 80 nM, respectively and with a good selectivity relative to MACs, although it is obviously difficult to obtain highly specific competitive inhibitors of an ATP-utilizing enzyme. Interestingly these results showed that EF would have a preference for the cytosine base unlike other adenylyl cyclases, which would allow better selectivity and potency [35,39]. No *in vivo* studies have been reported yet with these nucleotides analogs that might require further engineering to be rendered cell permeable.

#### 1.6.2. Inhibitors Targeting the EF-CaM Interaction

As EF is essentially inactive in the absence of CaM, preventing its interaction with its activator represents an efficient and potentially more selective approach to inhibit this enzyme. Yet, designing small molecule able to efficiently inhibit protein-protein interaction still remains a challenging task, and there are to date only two examples of inhibitors that act by preventing CaM binding and therefore EF activation. The first inhibitor of that class was identified by Tang’s group by screening a library with a combination of cell-based assay and a protein binding-based screen [36]. They first identified compounds that could block the EF-induced toxicity in a cell-based assay from a library of about 10,000 molecules. Selected molecules were further evaluated by testing their ability to prevent EF binding onto immobilized CaM with surface plasmon resonance (SPR). Among the 24 tested compounds, one, a 4-[4-(4-dichloro-phenyl)-thiazolylamino]-benzenesulfonamide, could bind to EF and prevent its activation by CaM. This compound, 10506-2A (Figure 2E), inhibited EF–CaM binding with an IC50 of 15 μM. A combination of fluorescence spectroscopy and photolabeling studies showed that the molecule targets the Helical domain of EF (*C*-terminal, Figure 1), which is involved in the binding of CaM. The 10506-2A inhibitor was selective for the EF-CaM interaction as it had no effect on two tested CaM-regulated MACs: calcineurin, a Ser/Thr phosphatase, and adenylyl cyclase 1 (mAC1). Yet, 10506-2A could also inhibit *B*. *pertussis* CyaA even though this toxin has a CaM-interacting region that is quite distinct from that of EF. As compound 10506-2A showed significant toxicity towards eukaryotic cells at 30–50 μM concentrations, Lee *et al*. [36] further characterized a series of 10506-2A analogs. They found one derivative, 4-[[4-(4-nitrophenyl)-2-thiazolyl] amino]-benzenesulfonamide (nitro10506-2A, Figure 2E) that was non-toxic to eukaryotic cell lines while it still blocked CaM binding to EF and prevented EF activation. The second family of inhibitors that prevent CaM-EF interaction [37] was designed through an *in silico* structure-based approach and will be described in details in the following sections.

### 1.7. Objectives

The purpose of the present manuscript is to show how computational analysis of the molecular mechanisms of EF activation by CaM led to a rational strategy to identify new inhibitors of the toxin. For that purpose, advanced computational biophysics techniques were applied to conceive and elaborate the novel design approach. The first ground for this strategy was established using calcium influence as a probe to understand the internal dynamics, energetics and thermodynamic stability of EF/CaM interaction. Then, the catalytic mechanism and the role of divalent ions were thoroughly investigated. Finally, a drug discovery strategy could be established by extensive exploration of conformational transition involved in EF activation and analysis of the associated cavity evolution. An allosteric site controlling CaM association and the concomitant EF activation could be identified. It was used to select small molecules by virtual screening for their suitability to bind to this site and block the CaM-induced EF activation. Several molecules selected *in silico* were characterized experimentally and a series of thiophen ureidoacid derivatives (TUA) were shown to inhibit EF activity. This validated the proposed allosteric site as a target for drug design.

## 2. Interplay between EF, Calmodulin and Calcium Ions

### 2.1. Structure of EF-CaM Complex

EF is composed of an *N*-terminal PA-binding domain (residues 1 to 291), a catalytic core domain (residues 292 to 622), and a *C*-terminal helical domain (Hel; residues 660 to 767). In the EF-CaM complex, CaM is inserted between the catalytic core and the helical domain of EF (Figure 1A). The catalytic core of EF is subdivided in two sub-domains: CA (residues 292–349 and 490–622, *in marine*) and CB (residues 350–489, *in purple*). Three loops, called Switches A, B, and C, or SA, SB and SC reorganize and assemble upon CaM binding to form and stabilize the active conformation of the catalytic site at the CA–CB interface (catalytic residues *in licorice*). The switch A region (domain CA, residues 502–551, *in cyan*) is in direct contact with the *C*-terminal lobe of CaM in the complex. Switch B (domain CA, residues 578–591, *in magenta*) adopts an active conformation forming a wall of the active site. Switch C (residues 630–659, *in red*) embraces CaM and maintains Switch B in place. Switch C also connects the catalytic core to the helical domain, Hel (*in wheat color*). The Hel domain is formed by four helices and directly interacts with the *N*-terminal lobe of CaM.

CaM is a small and very flexible protein bearing four calcium binding sites (Figure 1B). Each binding site is formed by a helix–loop–helix motif called “EF-hand”. The four binding sites are organized in pairs in two globular lobes, N-CaM and C-CaM, which are connected by a flexible inter-linker. Alpha-helices A, B, C, and D form the N-CaM domain, while helices E, F, G, and H form C-CaM. A fraction of helices D and E flanking their junction form an inter-linker region. The calcium-binding loops are located between helices A and B for site S1, helices C and D for site S2, helices E and F for site S3, and helices G and H for site S4.

The influence of calcium binding on CaM conformation and properties has been largely documented [40,41,42,43,44]. The α-helices forming CaM “EF-hand” motifs open upon calcium binding. This opening exposes hydrophobic patches [30], which can then promote interactions with amphipatic helical peptidic segments that commonly constitute the CaM-interacting sites of most CaM target enzymes. In the EF-CaM complex, C-CaM adopts an open conformation with exposed hydrophobic patches, whereas N-CaM adopts a closed conformation (Figure 1B).

### 2.2. Calcium Probes the Modulation of EF Activity by CaM

Calcium concentration has also a strong effect on EF-CaM complex, which could, to a certain extent, be considered as a ternary complex, involving EF, CaM and calcium ions. The affinity of EF for CaM has been shown to depend on calcium binding [24]. The complex appeared most stable with 2 Ca^2+^ bound in titration assays. The complex with 4 Ca^2+^ was marginally formed, and no EF-CaM complex could be observed in the absence of calcium. Calcium ions first bind with high affinity to sites S3 and S4 of the *C*-terminal CaM lobe (C-CaM). Then, in the *N*-terminal CaM lobe (N-CaM), sites S1 and S2 are progressively populated with increasing concentration of calcium ions. The modulation of the EF/CaM interaction by calcium provides a useful probe to further analyze the biophysical ground of EF-CaM complex formation and stability.

The interactions at play in the EF-CaM complex were characterized by molecular dynamics (MD) simulations performed with different numbers of bound Ca^2+^ ions. Following the experimental observations of [24], the number of calcium ions was modified *in silico* by removing or, on the contrary, adding two calcium ions to the 2 Ca^2+^-loaded complex to model the calcium-induced changes in EF and CaM conformations. The bending of CaM central linker observed with 0 and 4 Ca^2+^ ions suggested [45] that CaM acts as a spring. Its stiffness and opening would be tuned by calcium binding to optimally place EF Hel and CA domains and fold Switches A, B and C in their catalytically competent conformations.

### 2.3. Calcium Signal Propagation throughout EF-CaM Residue Network

As stated in the introduction, despite the important role of calcium, crystallographic structures of the EF-CaM complex with different packing geometries and different numbers of bound calcium ions do not display major variations [29]. Nonetheless, the Hel domain adopts slightly different conformations at the interface with CaM in the two sets of EF-CaM structures (Drum *et al.* 2002 [23] and Shen *et al.* 2005 [29]). These slight structural variations reflect subtle energetic equilibria and transfers arising from conformational and local flexibility modulation [46,47,48].

**Figure 3:**
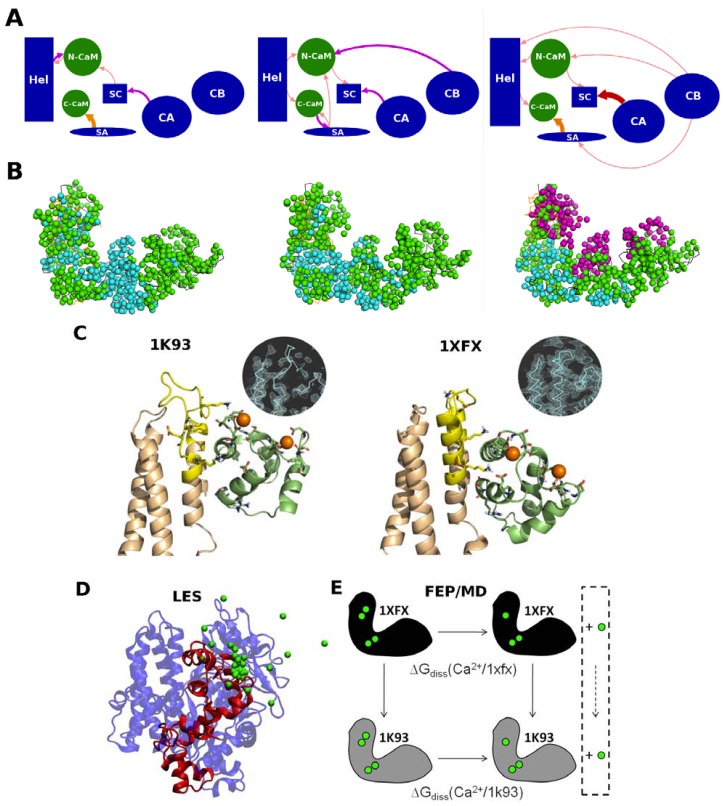
Calcium effect on the EF-CaM complex.Energetical influences and dynamical correlations computed in the EF-CaM complex. (**A**) the energetical influences between the different domains of EF (in blue) and CaM (in green) are drawn as arrows whose color and thickness indicate the intensities of the influences, as seen by MMPBSA energy dependency maps [49]; (**B**) the residues of the EF-CaM complex are represented by their C-α atoms and colored in cyan when their dynamical correlations are low (<0.5), in green when their correlations are mean (0.5–0.6) and in magenta when their correlations are high (>0.6). In the 2 Ca^2+^ bound complex (in the **middle**), the energetical influences are located in the vicinity of the EF/CaM interface and the most residue are correlated. Removal of calcium (on the **left**) reduces the energetical influences and a region of uncorrelated residues appears at the interface between the two proteins. Upon calcium addition (on the **right**), new energetical influences are observed even at long distances, and three sets of highly correlated residues were observed; (**C**) Interface between the Hel domain of EF and N-CaM in the EF-CaM complex. The conformations taken after 8 ns of MD simulations starting from 1K93 (on the **left**, chains C and F) and 1XFX (on the **right**, chains A and O) are represented in cartoon. EF Hel domain is colored in wheat with the inter L–M loop highlighted in yellow. N-CaM is colored in green lime. Residues involved in the EF/CaM interaction and residues forming CaM calcium binding sites S1 and S2 are drawn in sticks. Calcium ions are displayed as orange spheres. The inserts represent the electron density maps of the starting crystallographic structures, contoured at 1.10 s; (**D**) Principles of locally enhanced sampling (LES) methods; a snapshot from the LES simulations (protein in cartoons, ions in green spheres): to enhance the probability of calcium dissociation events, one Ca^2+^ ion is replicated in multiple copies; (**E**) Principles of free energy perturbation (FEP). The thermodynamic cycle used in the FEP method to compare calcium dissociation free energies between two protein conformational states (1k93-4Ca and 1xfx-4Ca complexes).

The propagation of the calcium signal through the residue network of EF-CaM was modeled using several recently developed approaches [49]: the generalized correlation [50], the local feature analysis [51], and the energy dependency maps [52]. The dynamical and energetical contributions at play within the complex were analyzed in details and compared in the different calcium level conditions. This analysis showed that the arrangement of the domains of the two partners in the complex can be visualized as a “house-of-cards” assembly of dependent sub-domains. In that assembly, CA, CB end Hel domains of EF act as foundation cards and the other structural elements, N-CaM, C-CaM, SA and SC, assemble on top with ranks in interaction strength that are shuffled by calcium removal or addition (Figure 3A,B). The most stable complex, with two bound calcium ions, corresponded to balanced interactions and influences between the domains of the complex. This molecular modeling investigation can be paralleled with the transmission of conformational signal [48,53,54] in elastic network models [46,47,55].

### 2.4. Interplay between EF/CaM and Ca/CaM

Energy dependency maps showed that the interface between EF and CaM is central to the architecture of the complex. This interface was explored in more details by recording two molecular dynamics trajectories starting from two different crystallographic structures of EF-CaM that represented two micro-states [56]. One trajectory was started from the 2Ca^2+^-loaded EF-CaM crystallographic structure (1K93 [23]) in which two additional calcium ions were added. The other trajectory was started from a crystallographic structure already containing four calcium ions (1XFX [29]).

The electronic densities observed in 1K93 and 1XFX displayed different flexibility and backbone conformations of the inter L-M loop of the Hel domain of EF, at the interface with N-CaM (Figure 3B,C). Based on this observation, it was supposed that the lower affinity of the N-CaM calcium binding sites S1 and S2 in the complex could be explained by conformational constraints induced by the Hel domain. This hypothesis was confirmed by molecular dynamics analyses [56], which highlighted the interplay between the constraint exerted by the Hel domain and the affinity of the calcium sites. Indeed, when the first “EF-hand” of CaM was more firmly attached to EF (simulation starting from 1XFX), the coordination of the Ca^2+^ ions was looser in the calcium binding loops of sites S1 and S2 than in the other simulation (starting from 1K93) (Figure 3C). The affinities of these sites for the calcium were also evaluated [56] with Local Enhanced Sampling (LES, or multiple copies) simulation and with FEP/MD simulations (Figure 3C) and were found to correlate with the Ca^2+^ ions coordination quality (number of interactions and their recorded distances along the trajectories).

Computational biophysics analysis of EF/CaM/Ca^2+^ interactions has revealed that the interface between the Hel and CA domains is the most sensitive part of the EF-CaM complex to the binding or removal of calcium on CaM. This direct influence of the number of bound calcium ions on EF activation [25] suggests that this sensitive region, which notably involves SA and SC, should be a favorable target locus for allosteric EF inhibitors candidates.

## 3. Catalytic Properties

**Figure 4:**
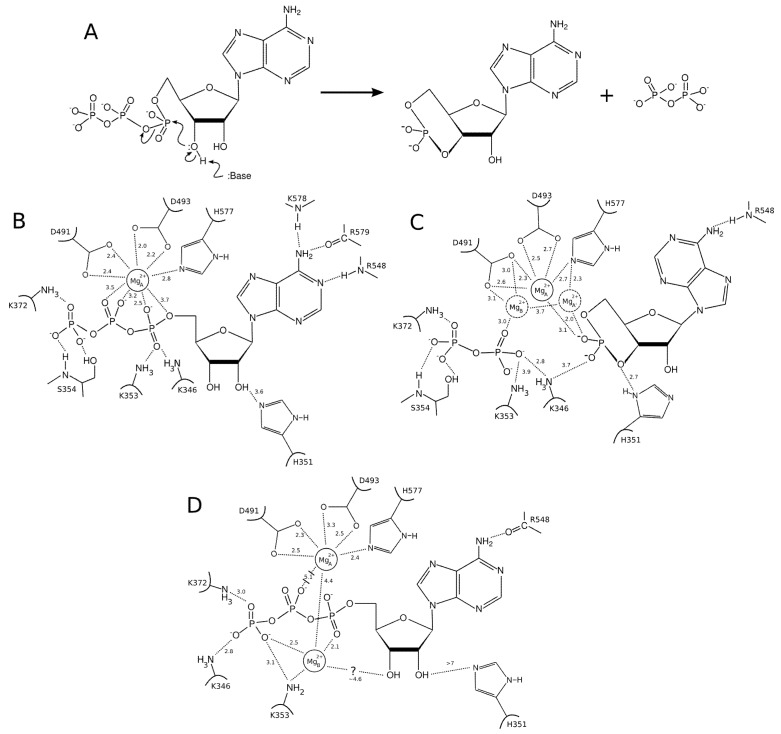
ATP cyclization mechanism. The arrangement of the active site suggested by crystallographic structures with (**A**) the basic cyclization reaction; (**B**) substrate and one metal ion (based on PDB structure 1K90); (**C**) products and either one and two metal ions (based on PDB 1SK6); and (**D**) the substrate and two metal ions (based on PDB 1/XFV). For clarity, all ions are represented as Mg^2+^, the actual catalytic ion, and the substrate analogs are shown as restored to ATP.

EF is an adenylyl cyclase turning ATP into cyclic-AMP (cAMP) more efficiently than MACs, as displayed in Figure 4A. It was also reported recently to have cytidylyl cyclase (CC) and uridylyl cyclase (UC) activities [2]. The canonical mechanism of these cyclizations involves the nucleophilic attack of ribose hydroxyl in position 3' to the Phosphorous of the α-phosphate group. This attack can be catalyzed by, first, the abstraction of the proton attached to hydroxyl in position 3' and, second, the stabilization of the highly charged pyrophosphate (PPi) group in formation [57,58]. Adenylyl cyclases accelerate the reaction by both mechanisms. The proton is believed to be removed by some proximal Lewis base, which are suggested, for example, to be aspartate or asparagine residues in MACs [58,59]. Stabilization of the leaving PPi group is achieved by binding to metal ions and cationic residues (lysines and arginines) [60]. Crystallographic structures have provided detailed pictures of the molecular arrangement of these constituents. This is especially the case for the active site and the number and position of metal ions in MACs. However, analysis of EF crystallographic structures raised unexpected questions on the catalytic site definition, which suggest that the details of the mechanism diverge from that of MACs.

### 3.1. EF Catalytic Site Structures

EF catalytic site is located between CA and CB domains, and the ATP binding pocket includes residues from six segments of the protein (Figure 1, [22]).

EF crystallographic structures were determined with different molecules bound to the catalytic site, different substrate conformations and different cationic binding modes [23,25,28,29]. These structures brought information about two different states of the catalytic site: (i) the state before the enzymatic reaction, with the ATP substrate analog 3'-deoxy-ATP, for which two structures have been determined (1XFV, 1K90); and (ii) the state after the reaction with the products cAMP and PPi, for which one structure is available (1SK6). As these structures correspond to different functional states, the position of the ions should reflect different binding properties. Furthermore, as various ion-binding modes were isolated for each state, their roles in substrate binding, catalysis and product release remained to be clarified, especially considering that the reaction involves highly charged groups. Noteworthy, 1XFV is the only structure obtained with Mg^2+^ ions, but at a concentration, which inhibits the reaction [25]. A substrate analog, 3'-deoxy-ATP, which cannot undertake the reaction towards products, was used instead of ATP for the reactant form. Structure 1SK6 was obtained with the highly charged Yb^3+^ which might have allowed trapping of the products in the active site. Hence, the details of these structures may differ from the functional states of the protein. Figure 4B,D depict the arrangement the substrates, ions and key residues in the active site, inferred from available crystallographic structures by substitution of ions and substrate with Mg^2+^ and/or ATP when necessary.

The first solved structure [23], corresponding to Figure 4B displays the substrate with only one metal ion in the active site. It was firmly attached both to the enzyme (through residues D491, D493 and H577) and to ATP phosphate groups. This differed from the consensual binding of two Mg^2+^ ions found in MACs and suggested differences in the detailed catalytic mechanisms. Nevertheless, the substrate position in that structure is similar to that of MACs.

The arrangement of the products bound to EF in presence of Yb^3+^, raised additional questions about the catalytic mechanism (Figure 4C, [28]). The electronic density suggested possible binding of either one or two ions in the active site. The one-ion binding mode is similar to the substrate-bound model previously described, with coordination by D491, D493 and H577, and interaction with PPi and cAMP phosphate oxygens (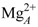 ion in Figure 4C). By contrast, the two-metal-ion binding mode suggests a weaker binding of the ions to the protein; one being mostly coordinated by D491 and PPi and the other mostly coordinated by H577 and cAMP. This arrangement resembles the two-metal-ion binding mode of MACs [60,61].

Finally, an experimental structure of the EF-ligand complex with two Mg^2+^ ions, the actual catalytic ions, was solved [29]. This suggests that EF could adopt the consensual MAC active site arrangement. However, as will be discussed below, the position of the ions in that structure (Figure 4D) seems rather resulting from a crystallographic artifact than a functional arrangement. Indeed, 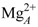 is coordinated by the protein in a single-ion mode like in Figure 4B, whereas the other ion, 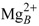 is mostly coordinated by ATP and makes poor contacts with the protein (Figure 4D).

### 3.2. Insights from Computational Modeling

Some experimental evidences suggest that EF and MACs might operate with different mechanisms. First, EF has a cAMP production rate of 1–2000 s^−1^, much higher than that of MACs. Second, the Mg^2+^ concentrations used to solve the two-Mg^2+^ structure 1XFV inhibit the catalytic reaction [25]. Thus, its relevance to interpret the catalytic mechanism is questionable. Third, comparative analysis of MAC and EF structures with two ions shows different substrate conformations and ions positions [61]. Hence, despite the same number of ions in the active sites of MACs and EF, inferring a conservation of the mechanism would be an oversimplification. Last but not least, the structure with a substrate conformation resembling to that of MACs was obtained with a single ion (Figure 4A), and is the one that appears to be compatible with the structure of the bound products [61].

Extensive molecular dynamics simulations and modeling have been performed [61,62] to analyze the relevance of each binding mode with respect to association, dissociation and affinity of the substrate and products.

Conventional Molecular Dynamics (MD) simulations of ATP and products bound to the active site were performed first [61]. These simulations suggested that: (1) The single ion in 1K90 (Figure 4B) is very effective in bridging the interaction of the phosphate tail of ATP to the active site residues, with strong interaction energies with both of them; (2) The ions in the two-ion binding mode (Figure 4D) are not as effective for ATP binding, because 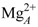 ion is tightly attached to the protein, but loosely to ATP, while 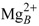 is essentially only coordinated by the substrate. Accordingly, ATP displays a larger mobility and flexibility in the active site of this conformation in MD relative to the one-ion binding mode; (3) The presence of two-ions in the product-bound active site firmly attach PPi and cAMP (Figure 4C). The global effect of this strong binding is ambiguous, as it may accelerate the reaction by reducing the transition state energy, but it could also slow down the release of products and thus the reaction. These simulations support that structure 1XFV (substrate and two-ions) would not correspond to a functional state.

Substrate, products and ion association and dissociation have large activation barriers and occur in too long time-scales for conventional MD. Hence, to analyze the effect of their conformation on their associations and dissociations in details, non-conventional MD techniques were used [62]. Locally Enhanced Sampling (LES) probes possible dissociation routes for substrates and products, by increasing the number of sampled ligand conformations and by reducing their interactions with the protein. The LES dissociation routes were then studied in details with regular interaction potential by Steered Molecular Dynamics (SMD) simulations.

ATP dissociates (and putatively associates) by diffusing in a wide opening between Switch C and α-Helix D, while the products, PPi and cAMP, dissociate from the less opened active site through solvent-accessible channels in opposite directions [62]. These general trends are independent of the detailed binding modes within the precision of LES simulations. In agreement with conventional MD results, products dissociation was significantly hindered by the presence of two ions in the binding site, due to very specific, close-contact ion-phosphate interactions. Therefore, presence of two ions appeared unfavorable for product dissociation. An interpretation of the ions electronic density spanning different positions in the product-bound structure (Figure 4C) could be that they correspond to three different ion-binding modes, favoring the attachment of the substrate (central position), the dissociation of cAMP, or that of PPi respectively [62].

Interestingly, simulations indicate that the second ion, which is only coordinated by ATP in 1XFV structure, systematically dissociates with ATP, without requiring breaking ion-protein interactions. Thus, 1XFV structure may reveal a transient state in which ATP would reach the active site with the second ion. Noticeably, the dissociation of ATP is easier from 1XFV structure than from that of 1K90 where the single ion, tightly bound to the protein and ATP, provides rigidity (conventional MD [61], and Figure 4B).

From the analysis of crystallographic models and simulations, a plausible model for EF function could be the following: (i) The association of Mg^2+^ bound ATP to a one-ion catalytic site, in a conformation resembling that of structure 1K90; (ii) The presence of two Mg^2+^ ions in the catalytic site would induce a 1XFV like conformation, which would reshuffle and lead to the expulsion of the 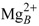 like ion, thus restoring a 1K90 like conformation competent for catalysis (Figure 4B,D). This reshuffling of Mg^2+^ ions in the active site may favor transient catalytic competent conformers of ATP [61]. Indeed, paradoxically, the 1K90 binding mode resembles to that found in MACs structure despite the presence of one ion only. Alternatively, Mg^2+^ free ATP molecules, present in low concentration could also enter the site, directly leading to the 1K90 like conformation. After (iii) cyclization; (iv) the dissociation of products could be facilitated by reshuffling of Mg^2+^ ions within their multiple possible positions and the entry of new Mg^2+^ ions to replace those that could have left with one of the products. This mechanism, based on a one-ion catalytic step with possible transient presence of two ions would be different from that of MACs. They suggest a rather plastic catalytic site allowing Mg^2+^ reshuffling. Obviously, further experiments and simulations are necessary to validate or refute this mechanism.

Other questions concerning the catalytic mechanism remain. Is His351 pulling the 3' hydroxyl proton out as a Lewis base (Figure 4B–D)? Experimental evidences are not conclusive: this residue may be found at distances up to 7 Å from the hydroxyl groups of ATP (in 1XFV), thus not allowing the formation of an hydrogen bond with hydroxyl in position 3' (Figure 4D, [29]). Experiments clearly show that His351 is fundamental for EF activity, but its role remains undetermined, as its substitution by other potential Lewis bases (as Asn) do not completely restore the enzymatic efficiency [63]. It has been proposed recently that Mn^2+^ could act as a physiological ion for ACs together with or in place of Mg^2+^ [64,65]. Mn^2+^ was found to occupy the two-ions binding sites of MACs with affinities different form that of Mg^2+^ [60]. The suggested roles of Mn^2+^ could challenge the current models of EF action mechanism. Hence, at this stage, many factors are still unknown, and it is difficult to identify the binding mode and substrate conformation actually involved in the reaction path and the transition state. The absence of a precise definition of the mechanism makes the analysis of the reaction difficult at electronic level since multiple hypotheses would have to be explored.

## 4. Conformational Transition and Inhibitors Discovery

### 4.1. Challenges in Rational Inhibitor Design

The rational design of new biomolecular inhibitors faces several challenges: (1) Identify a protein and a specific pocket or cavity at its surface to exert an effective therapeutic effect; (2) Search for ligands that bind with sufficiently high affinity to such a cavity to implement actual inhibition in physiological conditions; (3) Discover diverse chemical scaffolds to overcome drug design adversity, due, for instance, to various sources of attrition in drug development or to the apparition of drug resistance.

A straightforward approach to search for inhibitors is to target active sites or sites associated with a known effector. However, such a strategy is prone to identification of inhibitors resembling already known ones. For example, one known inhibitor of EF is the metabolite of adefovir, an ATP analogue, which binds the catalytic site with high affinity (see above, [26]). Although adefovir has been approved for the treatment of hepatitis B, it displays toxicity at high doses. Actually, the high degree of conservation of ATP-binding sites limits the selectivity of the inhibitors that target such sites and the high cellular concentration of ATP (around 5 mM, [66]) diminishes their efficacy.

As a consequence, any drug design strategy targeting the active site of EF bears a significant risk of designing promiscuous ATP analogues that will provoke detrimental side effects. In addition, as shown in section “Catalytic properties”, available structures do not provide an unequivocal catalytic mechanism nor a simple ion-binding pattern. Theses mechanistic uncertainties suggest an even stronger risk associated with the targeting of EF catalytic site in a rational drug design approach.

The question is then how can we identify another type of inhibitory site? Searching for possible inhibition mechanism, we can consider that it can operate either statically, by competition or by impeding the binding of functional partner, or, alternatively, dynamically, through allostery, which can also involve alteration of partner binding. The first class of static inhibition, especially competition, is rather classical, and it seems that exploiting the second dynamical category offers a larger number of opportunities. This second route appears promising, but requires a method to identify a novel-binding site at the surface of the protein, in which a small molecule could block the targeted activity upon binding. Noteworthy, use of a yet unexploited site would create ideal opportunities to design innovative molecules. Hence, targeting EF site different from the catalytic pocket appears as an attractive and valuable alternative, which is worth the attempt.

### 4.2. Design Strategy to Target EF Activation Mechanism

Identification of a yet unexploited inhibitory site is a motivating challenge. Nonetheless, operating inhibition that exploits a dynamical mechanism requires technologies to determine relevant locations at the surface of the target protein. Identifying the most promising target sites requires a deep understanding of the function and activation/deactivation molecular mechanisms to identify the essential motions as well as the associated cavity evolution. Indeed, the target site should directly or indirectly be involved in the function, but it should also be sensitive and responsive enough to the binding of a small compound to exert an effect.

**Figure 5:**
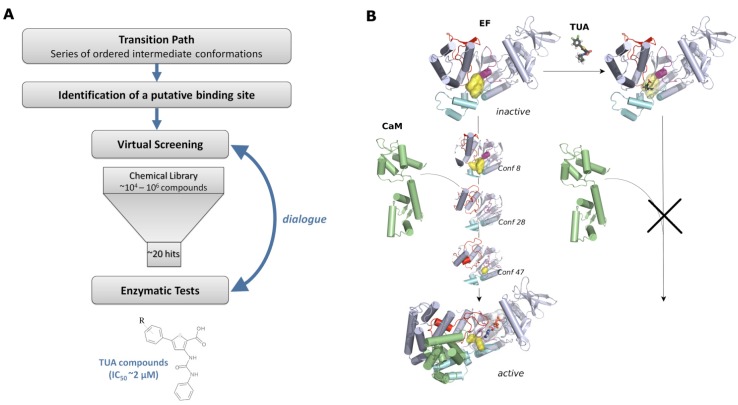
Use of EF conformational transition to search for allosteric inhibitors.(**A**) Workflow of the inhibitor identification; (**B**) Rational structural basis for an inhibition established by mean of an allosteric interaction. EF and CaM are drawn in cartoon and the TUA compound in licorice. CaM is colored in lime green, and the SABC pocket in yellow.

The outline of the rational strategy used to identify new inhibitors of the EF toxin, and which could in principle be employed for any target, is given on Figure 5A: (1) a transition path was determined that consisted of a series of ordered intermediate conformations between the protein inactive and active forms; (2) a putative binding site was then identified by analyzing the evolution of cavities along the path; (3) virtual screening was performed on the chosen pocket and about 20 hits were selected and tested by *in vitro* experiments; (4) this led to the discovery of a new family of inhibitors, sharing a common thiophen ureioacids scaffold. This overall strategy is described in more details in the following sections.

### 4.3. Identification of the Potential Binding Site

EF undergoes a very large and complex structural transition whilst it is activated upon binding of CaM. In the presence of CaM, the helical domain is subjected to a translation of 15 Å with a rotation of 30°, and several loops are reorganized to form the catalytically competent ATP site. These functional motions would have been difficult if not impossible to isolate/observe experimentally and advanced molecular modeling techniques were used to build plausible intermediate states. Hence, the conformational transition was described *in silico* by the Path Optimization and Exploration, POE, approach [37] developed to extend the power of the conjugate-peak algorithm [67,68] by iteratively identifying transitional shortcuts and reassembling them in a continuous, low energy and short length path.

Analysis of the cavities at the surface of the protein during this transition with in-house programs and ICM PocketFinder [69], covered, among others, already known cavities such as the catalytic site and cavities in the CB and Hel domains respectively [26,36].

It also pointed out a cavity displaying a strong shrinkage in the early stages of the activation transition path. This cavity was located mostly between the Switches A, B and C and was, thus, called the SABC pocket (Figure 5B). This pocket located on a side of the central region had never been reported as a site, which could be used to inhibit the toxin.

As shown in section “Interplay between EF, calmodulin and calcium ions” the region connecting the domains CA and Hel appears particularly sensitive to the calcium level (Figure 3). These analyses also highlighted the importance of the central region of EF, namely Switches A, B and C, during the activation process. Consequently, the SABC pocket appears indeed as a favorable putative site to target in order to block EF activation by trapping the protein in an intermediate inactive conformation (Figure 5B).

### 4.4. Identification of a New Family of Inhibitors

Once the target site was identified, virtual compound libraries were screened on the initial step conformations of the activation path model to select binder candidates. Intermediate conformations (labeled on Figure 5B) were used to perform an additional virtual screening against candidate compounds that could be compatible with activation. From a short list of about 30 candidates, 18 were available from a library of chemical entities of the French CNRS (Chimiothèque Nationale). The identified inhibitors were issued from the chemical library of the CERMN (Centre d’Etudes et de Recherche sur le Médicament de Normandie), developed using a program of parallel synthesis [70,71] to produce a library of 1140 thiophen derivatives with potential pharmacological properties. Their inhibitory activity was characterized experimentally with an *in vitro* enzymatic assay based on the detection of inorganic pyrophosphate released upon ATP conversion into cAMP [37].

Among those 18 compounds, a series of six highly related compounds, sharing a common thiophen ureidoacid scaffold (TUA) with different substituting groups, displayed a significant inhibitory profile. One of the compounds, a dichloro-TUA exhibited a clear inhibition at 10 μM (80% inhibition) with an estimated *IC_50_* of 2–3 μM.

Interestingly, those compounds exerted inhibition on the moderately homologous *B. pertussis* toxin, CyaA. Despite an overall low homology (25% identity in the catalytic domains), a pocket sharing significant identity and homology (40 and 80% respectively) with the SABC pocket of EF is present in the corresponding region of CyaA. Additionally, the compounds appeared more effective on EF before activation by CaM than after, showing obviously that the inhibition did not operate by competition on the active site.

A systematic cavity analysis performed on representative conformations extracted from the MD trajectories of the EF-CaM complex, with 0, 2 or 4 Ca^2+^ bound on CaM showed [37] that larger SABC pocket volumes correlated with a collapse of the catalytic site, thus providing additional insight on possible allosteric influences at the atomic level.

As a conclusion, ligand binding to SABC proved to impact activity through an allosteric effect. Allosteric inhibitor design is particularly interesting, as it provides opportunities to target a protein function by means of ligands that bind to sites distinct from the catalytic pocket. Noteworthy, it can also be used on non-enzymatic proteins. In the case of EF, since the catalytic site binds ATP, design of allosteric ligands avoids the conception of molecules that could interfere with ATP-binding cellular enzymes, and hence, problems of inhibitory promiscuity, side-effects, and general toxicity. Interestingly, a similar situation is encountered for kinases, a major class of pharmaceutical targets binding ATP, for which allosteric inhibitors provide opportunities to improve selectivity [72].

## 5. Conclusions

Molecular modeling is an essential tool to build hypotheses on biomolecular function. This goes through the building of structural and mechanistic models of the involved biomolecular assemblies. It can be used to further analyze and understand how molecular systems work, for example through the design of strategies to probe protein activity. Along this line, molecular modeling can also be used to design ways to operate on biological and/or living materials, as, among many possibilities, through the design of an inhibitor.

The large amount of available structural data on EF, owing to investment of Wei-Jen Tang’s team, has been essential for this approach. More specifically, access to both inactive and active forms of EF structure has been an inestimable piece of information.

As EF undergoes a large conformational transition to produce the EF/CaM active complex, it appeared as an excellent model to elaborate such a strategy. This idea was supported by the molecular modeling analysis of the mutual influences and plasticity of the partners in the context of the conformational transition.

One application of molecular modeling having practicable and valuable outcomes is to provide bases for the rational design of strategies to identify new types of inhibitors, especially allosteric ones, which are of major interest for the pharmaceutical industry. Various classes of targets can be cited, among which, GPCR and kinases [73,74].

Molecular modeling hypotheses and models allow for the elaboration of ambitious and innovative strategies, but the latter heavily relies on the quality of those models and hypotheses. Therefore, model validation is an essential step for such an approach. The biochemical validation of an inhibitor among rational design candidates appears as a rewarding validation of the long chain of hypotheses that was built to elaborate the design strategy. Conversely, newly identified inhibitors can be used to further probe the activity mechanism.
